# Miscellaneous

**Published:** 1892-12

**Authors:** 


### MISCELLANEOUS.

### INTELLIGENT DOGS.

This dog is worthy of an immortal spirit. The *News*, of Le Sueur, Minn., says : Sunday evening as the clouds began to gather and rain was imminent, Charles Harer, who lives on the hill, placed a board under an eaves of his barn to catch the water in case of rain. This board was slantingly placed and rested, at the lower end, on the edge of a half-barrel. Monday, the oldest of the Harer children, a girl about 9 years of age, took the youngest, an infant boy not yet a year old, out to play. She set him down on this inclined board and before she was aware of it the little fellow had rolled off into the half-barrel, which was filled with water from the heavy rain of the night before. Frightened, she ran to the house to call her mother who was in the cellar. When they returned, what was the mother's relief to see that their dog, a shepherd, had taken in the situation and grasped the little boy, who would otherwise have drowned, by the clothes on his back and held him aloof from the water until help came. Of course the parents feel very kindly to this dog now, and would scarcely part with him for any consideration. Carmichael, the artist, photographed him yesterday.

Mr. William Robsen writes the English *Fanciers' Gazette* as follows: I think the following anecdote of a dog will bear out the opinions of many who believe that animals are endowed with the faculty of reason :

I am a real lover of animals, and I am always glad to hear any anecdote which redounds to their credit, if it be authentic; so I am quite disposed to believe what a gentleman told me of his beautiful collie dog one day. I was stroking his silky black and tan coat, and admiring his large, affectionate, intelligent eyes, at the same time reading the name and address legibly engraved on his brazen collar. I said: Did this ever bring Scoti back to you? Only last week, said my friend, I lost him somewhere in Piccadilly. You know how much I rush about in hansom cabs, and Scoti always goes with me—we travel many miles in a week together in this way—but on this occasion I was walking, and missed him. Search was in vain. The crowd was great; traffic drowned the sound of my whistle, and after waiting a while and looking everywhere, I returned to my suburban home without my companion, sad and sorrowful, yet hoping that he might find his way back. In about two hours after my arrival a hansom cab drove up to the door, and out jumped Scoti. The cabman rang for fare, and thinking he had somehow captured the runaway, I inquired how and where he had found him. "Oh, sir," said cabby, "I didn't hail him at all; he hailed me. I was a-standing close by St. James Church a looking for a fare, when in jumps the dog. Like his impudence, says I; so I shouts through the winder, but he would not stir; so; I gets down and tries to pull him out and shows him my whip; but he sits as still as ever, and barks as much as to to say, 'Go on, old man.' As I seizes him by the collar, I reads the name and address. All right, says I. My fine gentleman settles himself with his head just a looking out, and I drive on till I stops at this gate, when out jumps my passenger a clearing the doors and walks in as though he had been a reg'lar fare." Need I say my friend gave the loquacious cabman a very irregular and liberal fare, and congratulated Scoti on his intelligence—be it instinct, reason, or whatever it may be that told him that hansom cabs had often taken him safely home. Who shall say dogs do not reason or reflect?

---

### A KING SEEKING A BRIDE.

Tablets found in Egypt at Tel-el-Amarna contain among other curious records the letters sent by a King of Egypt about 1500 B. C. to a King of Babylon denying that he had ill treated one Babylonian wife and asking for another. The *Pall Mall Gazette* gives the outline of some of those letters, which by Dr. Bezold's careful and scholarly translation now all those who run may read. Take the tablet of Nile mud, for instance, on which the scribe of Amenophis III. wrote the letter that was never dispatched to "Kallimma Sin, King of Karaduniyash, my brother." After a characteristically Eastern and ponderous beginning, in which good health is wished to the King and the King's wives, the government, horses and carriages, the Pharaoh tries to clear himself of the charge brought against him that he has not treated well one of his wives the sister of Kallimma Sin. "From the time when my father gave thee my sister to wife," the Babylonian King had written, "no man hath seen her, and none knoweth whether she be alive or dead." Now the King of Egypt has asked in marriage Sukharti ("the Little One"), Kallimma Sin's fair daughter. Hence these reproofs. The Egyptian thereupon challenged the Babylonian to send messengers who might convince themselves of the well-being of the wife whom "no man hath seen." The messengers come, but cannot, among the galaxy of Queens of Amenophis, identify the Babylonian Princess and Amenophis now writes in righteous indignation in the letter under consideration.

"Since thou sayest, My messengers cannot identify her, I answer, Then who can identify her? and I ask further, Why dost thou not send a wise man who might give thee a trustworthy account and describe to thee the comfort and the good health of thy sister here? Command, then, one of thy wise men to come and examine her household, and let him see for himself the honor in which she is held by the King."

After a great deal of haranguing, in which he of Mesopotamia asserts that his daughter Sukharti was "not beautiful," and after endless haggling over the loan of a quantity of gold, the marriage settlements are satisfactorily concluded, and Kallimma Sin writes to the King of Egypt: "If thou wilt write unto me she shall be brought unto thee."

Ten years after Amenophis III. had begun to reign, when, as it is stated on several large steatite scarabs, he had slain 102 lions with his own hand, he added another wife to his harem, Queen Thi, on whose tomb we read that she was a "royal daughter, royal sister, royal mother, royal wife, great lady, lady of the North and South," became the acknowledged Queen of Amenophis and of Egypt. Again and again the name of fair haired, blue eyed Queen Thi is mentioned in the Tel-el-Amarna tablets, and it also occurs frequently on the rings, vases, scarabs, and amulets of the reign of Amenophis III.

---

A Curious Eye.—The eye of the American buzzard, says the *Optician*, according to a descriptlon given by a taxidermist, must be a very unique arrangement. In effect it is practically as good as an opera glass. The bird can screw it in or out, so to speak, and fit it to the distance across which he wants to look at any object. The eyeball is surrounded by horny plates which move slightly on each other. The muscles at the back of the eye are so arranged that they can thrust the centre of the ball of the eye out in front, and the pressure of these horny plates keeps the whole eye from being pushed out also. Thus the eye assumes a cone-like appearance from the outside. By relaxing this pressure and exerting these muscles in the opposite direction the front of the eye can be made flat. By this means the buzzard can, while flying at a great height, sweep the ground with his telescope eye in search of carrion, and as he approaches the earth adjusts his sight so that he sees the desired object plainly, even when under his very beak.

The Only Good Hair Tonic.—Hair washes are generally more harmful than good. There is only one substance on the face of the earth that has any virtue as a tonic and that is rosemary oil, An essence made from rosemary oil greatly diluted in water is an excellent wash for hair that is becoming thin or falling out. It seems to revive and strengthen the scalp and stimulate the hair bulbs to renewed activity. All the other washes, more particularly those applications which contain grease of any kind, are far more hurtful than beneficial, since they clog up the scalp and prevent the natural nourishment being supplied to the hair.

### THE PAN-AMERICAN MEDICAL CONGRESS.

This body, which is to meet in Washington, D. C., in September, 1893, bids fair to be of great interest to the medical profession, particularly as a means of disseminating the most approved methods of medicine and surgery in their various departments.

### PREMIUM EXTRAORDINARY.

Having become throughly convinced of the harmless energy of Herba Vita, in preventing and eradicating many of the disorders common to all sections of this country, particularly those of a malarial tendency, we have concluded an arrangement with its proprietors, by which we are able to offer to every new or renewed subscriber to our Journal, in addition to other premiums, a 25 cent package of this valuable household remedy, which will be sent by mail post paid, to any given address on receipt of subscription, either direct or through the ordinary provided agencies.—HALL'S JOURNAL OF HEALTH.

